# Identification of markers that functionally define a quiescent multiple myeloma cell sub-population surviving bortezomib treatment

**DOI:** 10.1186/s12885-015-1460-1

**Published:** 2015-05-30

**Authors:** Alfred Adomako, Veronica Calvo, Noa Biran, Keren Osman, Ajai Chari, James C Paton, Adrienne W Paton, Kateri Moore, Denis M Schewe, Julio A Aguirre-Ghiso

**Affiliations:** 1 Division of Hematology and Oncology, Department of Medicine, Mount Sinai School of Medicine, New York, NY 10029 USA; 2 Department of Otolaryngology, Mount Sinai School of Medicine, New York, NY 10029 USA; 3 Tisch Cancer Institute, Mount Sinai School of Medicine, New York, NY 10029 USA; 4 Black Family Stem Cell Institute, Mount Sinai School of Medicine, New York, NY 10029 USA; 5 Research Centre for Infectious Diseases, School of Molecular and Biomedical Science, University of Adelaide, Adelaide, 5005 Australia

## Abstract

**Background:**

The mechanisms allowing residual multiple myeloma (MM) cells to persist after bortezomib (Bz) treatment remain unclear. We hypothesized that studying the biology of bortezomib-surviving cells may reveal markers to identify these cells and survival signals to target and kill residual MM cells.

**Methods:**

We used H2B-GFP label retention, biochemical tools and in vitro and in vivo experiments to characterize growth arrest and the unfolded protein responses in quiescent Bz-surviving cells. We also tested the effect of a demethylating agent, 5-Azacytidine, on Bz-induced quiescence and whether inhibiting the chaperone GRP78/BiP (henceforth GRP78) with a specific toxin induced apoptosis in Bz-surviving cells. Finally, we used MM patient samples to test whether GRP78 levels might associate with disease progression. Statistical analysis employed t-test and Mann-Whitney tests at a 95% confidence.

**Results:**

We report that Bz-surviving MM cells *in vitro* and *in vivo* enter quiescence characterized by p21^CIP1^ upregulation. Bz-surviving MM cells also downregulated CDK6, Ki67 and P-Rb. H2B-GFP label retention showed that Bz-surviving MM cells are either slow-cycling or deeply quiescent. The Bz-induced quiescence was stabilized by low dose (500nM) of 5-azacytidine (Aza) pre-treatment, which also potentiated the initial Bz-induced apoptosis. We also found that expression of GRP78, an unfolded protein response (UPR) survival factor, persisted in MM quiescent cells. Importantly, GRP78 downregulation using a specific SubAB bacterial toxin killed Bz-surviving MM cells. Finally, quantification of Grp78^high^/CD138+ MM cells from patients suggested that high levels correlated with progressive disease.

**Conclusions:**

We conclude that Bz-surviving MM cells display a GRP78^HIGH^/p21^HIGH^/CDK6^LOW^/P-Rb^LOW^ profile, and these markers may identify quiescent MM cells capable of fueling recurrences. We further conclude that Aza + Bz treatment of MM may represent a novel strategy to delay recurrences by enhancing Bz-induced apoptosis and quiescence stability.

**Electronic supplementary material:**

The online version of this article (doi:10.1186/s12885-015-1460-1) contains supplementary material, which is available to authorized users.

## Background

The overall survival of patients with multiple myeloma continues to improve, in large part due to proteasome inhibitors (PIs) and immunomodulatory agents [1, 2]. However, the majority of patients treated with these drugs inevitably relapse after variable remission periods [3]. Much effort has been spent in understanding how PIs induce pathways that regulate cell death during the acute treatment of these patients [4]. Similar effort has been placed in finding ways to maximize PI effectiveness and duration of response. However, less is known about the biology of residual MM cells that survive therapy, how to identify them, and how they persist after treatment [5, 6]. Currently, there are no universal criteria for identifying and tracking residual cells in MM patients in remission [7]. Understanding the biology and characteristics of MM residual disease, thus, represents a key avenue to prevent relapses.

PIs induce MM cell death by regulating several tumor cell intrinsic and stromal pathways [8]. Among these pathways, PIs are powerful activators of the unfolded protein response (UPR). This pathway has the ability to induce cell death but it also can induce growth arrest and survival as a first response to endoplasmic reticulum (ER) stress. We previously showed that acute exposure to bortezomib (Bz) treatment activated a canonical PERK-eIF2α-CHOP pathway that resulted in the majority of MM cells entering cell death [6]. However, MM cells surviving Bz treatment downregulated eIF2α phosphorylation, upregulated the survival chaperone BiP/GRP78 and entered a prolonged G_0_-G_1_ cell cycle arrest. Dephosphorylation of eIF2α in quiescent surviving MM cells was key for survival because inhibition of GADD34/PP1C, an eIF2α phosphatase, killed almost all surviving MM cells [6]. While these studies identified a survival mechanism for MM cells that persist after Bz treatment, they did not explain what cell cycle machinery components regulated the prolonged growth arrest and survival after Bz treatment. Further, the role of BiP/GRP78, an HSP70 family member for which inhibitors are in development [9], in Bz-surviving MM cells was also unknown.

Here, we show that MM cells that survive proteasome inhibitors display a GRP78^HIGH^/p21^HIGH^/CDK6^LOW^/P-Rb^LOW^ profile. We also provide preliminary evidence that higher levels of GRP78 detected in MM patient bone marrow biopsies may be present in patients with more aggressive disease and that GRP78 downregulation potentiated Bz killing. Thus, these markers may pinpoint quiescent MM cells with the ability to persist after treatment and sensitivity to Grp78 inhibition. We also show that apoptosis can be potentiated and quiescence extended by a sequential 5-azadeoxycitidine and Bz treatment. This drug combination schedule might represent a novel strategy to potentiate Bz efficacy in MM disease treatment.

## Methods

### Reagents, cell lines, tissue culture and quantitative reverse transcription-PCR

Antibodies: Anti-BiP/GRP78 [610979, BD]; Anti-CD138 [sc-5632, Santa Cruz]; Anti-Ki67 [9449, Cell Sig.]; Anti-P-Rb (Ser807/811) [8516, Cell Sig.]; Anti-P-Rb (Ser249/Thr252) [sc-377528, Santa Cruz]; Anti-p21 [2947, Cell Sig]; Alexa Fluor® 488 Goat Anti-Mouse, [A-11001; Invitrogen]; Alexa Fluor® 568 Goat Anti-Rabbit, [A-11008; Invitrogen]). Vectastain ABC kit and DAB peroxidase substrate kit was used for IHC developing [Vector lab]. Bortezomib (S1013, Selleck Chemicals) was used to treat RPMI8226 (CCL-155, ATCC) and U266 (TIB-196, ATCC) cells at 4 nmol/L or 8 nmol/L Bz for 24 h. The drug was removed by washing 3x with PBS and then re-plated in fresh medium (RPMI-1640 with 10% FBS). Cells were cultured according to ATCC recommendations. In 5-azacytidine (Aza) (A2385, Sigma) experiments, the cells were pre-treated for 4 days with 500 nmol/L Aza (and replaced every 48 h) before Bz treatment. Total RNA was extracted using Trizol. Primers used are in [Additional file 1: Table S1].

### Mouse xenograft studies

Institutional Animal Care and Use Committees (IACUC) at Mount Sinai School of Medicine (MSSM) approved all animal studies. Protocol ID: 11-0032PRYR1. ATCC-derived RPMI8226 and U266 cells were expanded and pulsed for 24 h with 8 nM Bz or DMSO vehicle control. Cells were then washed and viability was assessed by Trypan blue exclusion assay. Equal number of live (1 × 10^6^) cells was then resuspended in PBS with 50 % Matrigel (356231, BD), and injected s.c. into 4- to 6-week-old male NSG mice (Charles River). Tumor volumes were measured and calculated using the formula (*D* × *d*^2^)/2, where *D* is the longest and *d* is the shortest diameter. All points represent independent biological samples with error bars representing standard deviations and statistical significance determined using a Mann–Whitney test.

### Nuclear and chromatin extraction and western blots

After drug treatments, cells were washed with PBS and resuspended in 2 mL of Buffer A (10 mM HEPES pH = 7.9, 10 mM KCl, 1.5 mM MgCl2, 0.34M sucrose, 10 % glycerol) with 1 mM DTT, protease inhibitors, and 0.1 % Triton X-100 on ice for 7 min. The cells were then spun at 4,000 rpm and 4°C for 4 min. The pellets, containing the nuclear fractions, were resuspended in 300 μL of 2× Laemmli sample buffer per 10 × 10^6^ cells and then heated to 95°C for 10 min for western blotting. For chromatin fractions, the nuclear extracts were treated with “no salt buffer” (3 mM EDTA and 0.2 mM EGTA) before addition of 2× Laemmli sample buffer. For whole-cell lysates, cells were lysed for 30 min with lysis buffer containing 1 % Triton X-100, 50 mM Hepes, pH 7.5, 150 mM NaCl, 1 mM CaCl_2_, 1 mM MgCl_2_, 1 mM orthovanadate, 1 mM NaFl, and protease inhibitors. Western blots were performed as previously described [10] and imaged using Image Quant LAS (GE).

### Patient samples

Bone marrow aspirates (BMA) from multiple myeloma patients were collected in heparinized tubes following an Icahn School of Medicine Institutional Review Board approved protocol (Number: MSSM HS 10-00105). The BMAs were then subjected to density gradient centrifugation using Ficoll-Paque Plus (17-1440-02, GE). The isolated bone marrow mononuclear cells were then incubated with CD138 MicroBeads (130-090-503, Miltenyi) and separated using autoMACS separator (Miltenyi). CD138-positive cells were fixed and spun onto slides. To test for enrichment, mononuclear cells before and after separation were stained for CD138. RPMI8226 cells were stained as a positive control. The percentage of CD138-positive cells increased from about 8 % pre-separation to 97 % post-separation in all patients. The patient slides were stained with BiP/GRP78 primary antibody overnight and Alexa Fluor® 488 goat anti-mouse secondary antibody the next day. For controls, slides were stained with the secondary antibody alone. The slides were imaged using Leica DM6000 and quantified with ImageJ (NIH).

### Immunofluorescence (IF) and Immunohistochemistry (IHC)

For IF analysis of cytospins, cells were separated by density gradient centrifugation using Ficoll-Paque Plus after treatment to remove dead cells. The live cells were then fixed in 4 % paraformaldehyde in PBS (15 mins) and cyto-spun onto slides. Slides were then washed, permeablized using 0.5 % Triton X-100 and blocked for an hour with 3 % normal goat serum and 3 % BSA in PBS. The slides were then incubated with primary antibodies or diluent (1 % BSA in PBS) overnight at 4°C. After washing, the slides were incubated with secondary antibodies. The slides were then washed and mounted with Prolong gold anti-fade reagent with DAPI [P36931, Invitrogen]. Slides were images using a Leica DM5500B microscope and analyzed using MetaMorph®. For IHC analysis of tumors, tissues were fixed in 4 % PFA for 24 h, and then transferred to 70 % ethanol until processing for paraffin embedding and sectioning into 4-um-thick slices. Slides were deparaffinized and rehydrated through xylene and ethanol washes, and antigen unmasking was performed by heat-induced retrieval in citrate buffer. Quenching of endogenous peroxidase activity was done with 3 % H2O2. After blocking with 3 % normal goat serum in 3 % BSA/PBS for 1h, slides were incubated with primary antibodies overnight at 4°C. After washing, either fluorophor-conjugated secondary antibody was used and then mounted or an avidin/biotin peroxidase system was used and developed with peroxidase substrate kit [Vector lab]. In the latter case, VectaMount mounting media was used [Vector lab]. For quantification purposes, at least 10 randomly selected 20x fields were counted.

### Generation of the H2B-GFP tagged line and label retention assay

The Tet-inducible H2B-GFP construct was a kind gift from Dr. Kateri Moore [11]. The plasmid was transfected in 293T cells. Lentiviral particles were harvested from 293T cells and used to infect RPMI8226 cells. The infected cells were selected for stable expression using puromycin (1 ug/mL). Upon induction of H2B-GFP with doxycycline (1 ug/ml), high expressers were sorted using FACSAriaII (BD). For label retention experiments, the cells were induced with doxycycline for 6 days and released at the time of Bz treatment. Label retention was analyzed using FACS LSR Fortessa (BD). For viability assessment, Trypan blue exclusion assay was performed.

## Results

### Bortezomib-surviving MM cells display a CDK6^LOW^/p21^HIGH^ quiescent profile

**Fig. 1:**
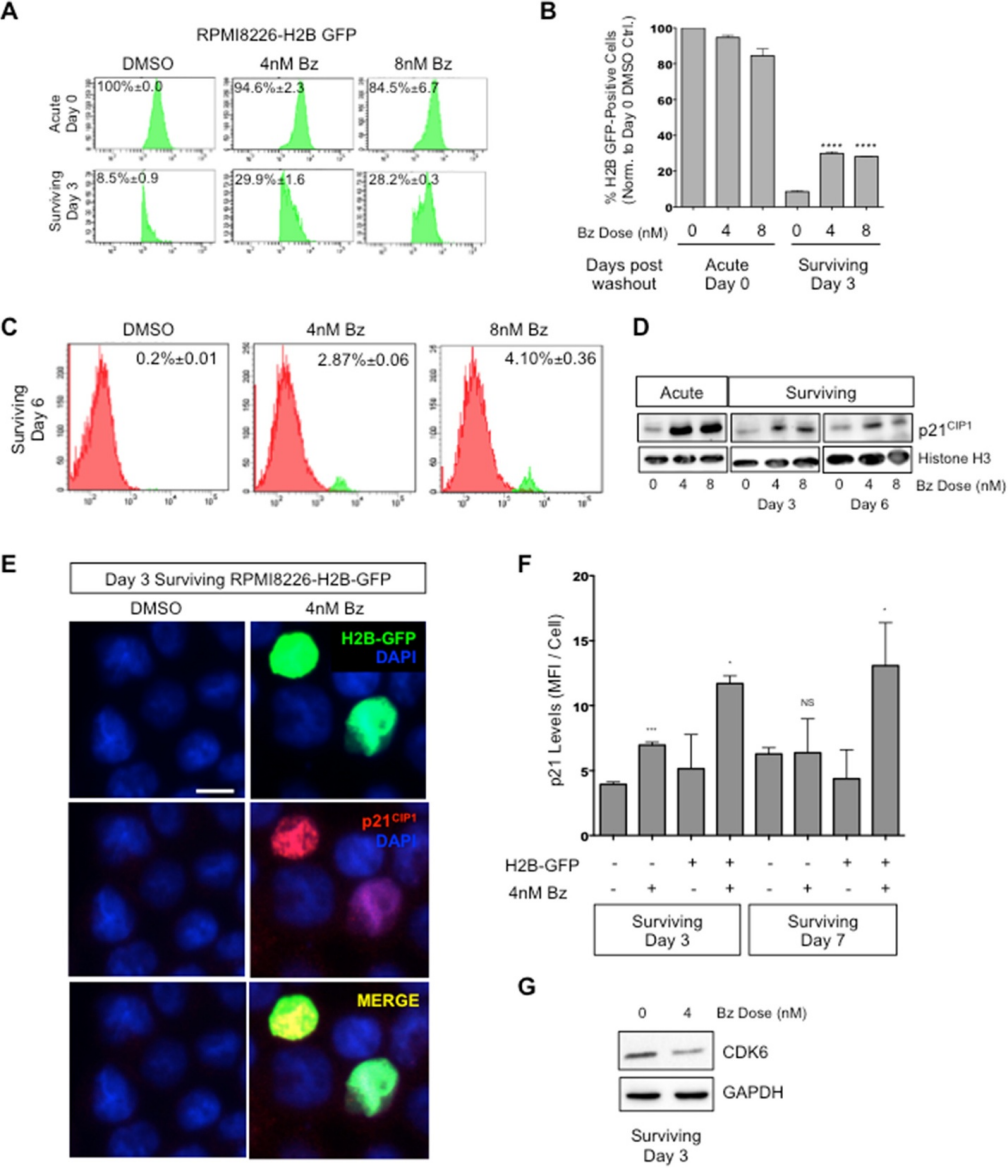
**a** Representative histogram of FACS analysis of H2B-GFP label retention in RPMI8226-Tet-H2B-GFP cells 0 and 3 days after a 24-h pulse with Bz (4 nM and 8 nM). Percentages = Percent of H2B-GFP^HIGH^ ± standard deviation. **b** Quantification plot of H2B-GFP^HIGH^ label retention. ****, *P* < 0.0001 comparing surviving day 3 DMSO to 4 nM Bz or 8 nM Bz, (unpaired *t* test). **c** Representative histogram of FACS analysis of H2B-GFP label retention 6 days after 24-h pulse with Bz. Percentage of cells was calculated using BD FACSDiva software (BD). **d** Western blots for p21^CIP1^ protein in nuclear extracts of cells surviving proteasome inhibition 0, 3 and 6 days after drug washout. Total Histone H3 was used as a loading control. **e** & **f** Detection and quantification of p21^CIP1^ in H2B-GFP^HIGH^ label-retaining cells at 3 and 7 days after drug washout by IF. Quantification was done using ImageJ. * p = 0.0361 comparing day 3 H2B-GFP-positive cells in DMSO vs. 4 nM Bz (unpaired *t* test). * p = 0.0475 comparing day 7 H2B-GFP-positive cells in DMSO vs. 4 nM Bz (unpaired *t* test). **g** Western blots for CDK6 protein in cells surviving proteasome inhibition 3 days after drug washout. GAPDH was used as a loading control. Scale bar =20 μm

We used a stable RPMI 8226 cell line virally transduced with a lentivirus containing a Tet-inducible H2B-GFP construct (see Methods). This inducible H2B-GFP label retention system allows marking the nucleosomes of cells by inducing the H2B-GFP transgene with Tetracycline. After de-induction (Tetracycline removal), only cells that do not divide and thus do not or slowly turnover their nucleosomes can be tracked as quiescent tumor cells for very long periods [12]. Tetracycline-treated RPMI-Tet-H2B-GFP cells (H2B-GFP^HIGH^) were washed, pulsed for 24 h with Bz (4 nM and 8 nM) and then followed for 3 days by FACS [Fig. 1a-b]. Using gates that detected viable H2B-GFP^HIGH^ label retaining cells, we found that while DMSO-treated cells lost most of their labeling within 3 days (~8.5 % +/-0.9), ~30 % of the Bz-treated cells continued to retain high H2B-GFP labeling (~3.6 fold more in the MFI >10^3^) [Fig. 1a-b]. By 6 days, while label retention continued to decrease in control cells, Bz-surviving cells still displayed a 14- to 20-fold increase in H2B-GFP^HIGH^ cells [Fig. 1c]. U266 cells pulsed with an equivalent dose of the PI MG132 also remained quiescent up to 8-10 days before entering log-phase of proliferation [Additional file 2: Figure S1A]. In our previous study [6] and experiments here, we also used the proteasome inhibitor MG132 to show that the effects are due to proteasome inhibition and not some unspecific Bz effect. Using these two inhibitors, we reported previously that cells surviving Bz treatment are not irreversibly damaged and continuously entering apoptosis after drug washout, but rather entering a growth arrest that was measured using cell cycle profiling, P-Rb phosphorylation and label retention assays [6]. In addition, that the surviving quiescent fraction is viable is further supported by the detection of label retention of H2B-GFP [Fig. 1a].

Analysis of the viable quiescent Bz-surviving MM cells using immunofluorescence and western blot (nuclear fraction) showed that these cells were enriched for the cyclin-dependent kinase inhibitor p21^CIP1^ mRNA and nuclear protein [Fig. 1d-f]. The CDK inhibitors p15 and p16 mRNAs were also induced but p21^CIP1^ mRNA showed the strongest induction [Additional file 3: Figure S2E], which could be followed by Western blot and IF. This was observed at the end of the acute phase (Day 0) and at 3, 6 and 7 days after washout of the 24 h pulse of Bz [Fig. 1d-f]. Furthermore, H2B-GFP^HIGH^ cells expressed significantly more p21^CIP1^ in Bz-treated cells compared to DMSO controls [Fig. 1e-f]. Arguing for a G0-G1 arrest, p21^CIP1^ induction correlated with the downregulation of CDK6 protein as measured by Western blot [Fig. 1g] and with decreased levels of cyclin-D3 and CDK4 protein levels in proteasome inhibitor-pulsed cells [Additional file 2: Figure S1B]. This is in agreement with our data from [Fig. 1a-c] showing the existence of a deeply quiescent population in vitro.

**Fig. 2:**
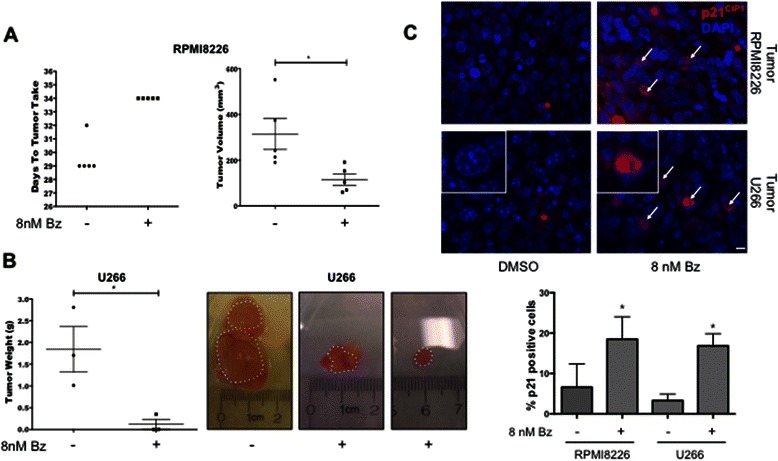
**a** Tumor latency and volume plot. Mice were injected with RPMI8226 cells that had been pulsed for 24 h with either DMSO (n = 5) or 8 nM Bz (n = 5). Palpable tumors were observed in DMSO treated at day 28 (none in 8 nM Bz) and measurable at day 29. Tumors were measurable in 8 nM Bz mice at day 34 suggesting a 5-day latency period until tumor growth. *p = 0.0245 comparing DMSO to 8 nM Bz mice at day 34 (unpaired *t* test). **b** Tumor weight plot of U266 cells treated as in [A], excised on day 86 after injection. *p = 0.0322 (unpaired *t* test). Representative images of tumors at day of sacrifice. Tumors were in some cases excised with surrounding subcutaneous tissue. Dotted lines indicate the margins of the tumors determined as best as possible by visual inspection. **c** IHC detection of p21^CIP1^ levels in tumors derived from RPMI8226 and U266 cells pulsed for 24 h with DMSO or 8 nM Bz (n = 3). Quantification of percentage positive nuclear p21^CIP1^ in tumor sections. *p = 0.05 comparing DMSO vs. 8 nM Bz (unpaired *t* test). Scale bar =25 μm. Insets show details of p21 negative (DMSO) and positive (8 nM Bz) cells

To determine whether Bz-surviving cells would also remain quiescent in vivo, RPMI8226 cells were pulsed for 24 h with Bz (8 nM or DMSO as control, n = 5 per group), washed and equal number of viable cells were injected into NSG mice. By Day 29-30, palpable tumors were detected in the DMSO group. The Bz pulse induced a delay of 2-5 days in tumor take and a significant difference in final tumor volume between DMSO and Bz mice [Fig. 2a]. A longer delay in tumor take was observed in mice injected with U266 cells, which also entered a longer quiescence in vitro [Additional file 2: Figure S1A]. By 80 days, there were palpable tumors in 5/10 of the mice injected with DMSO treated MM cells and no obvious palpable tumors in the 10 mice that received 8 nM Bz-treated cells. However, upon necropsy, we found that the mice injected with Bz-treated cells contained small dormant tumor nodules [Fig. 2b]. The dormant phenotype was tested to determine whether the in vivo growth suppression was due to increased cell death, quiescence or both. Sections of these tumors were stained for the apoptosis marker cleaved caspase-3 and the quiescence marker p21^CIP1^. This analysis revealed that the quiescence induction in Bz-surviving cells was also recapitulated in vivo. Bz-pulsed-RPMI8226- and -U266-derived tumors showed elevated levels of p21 nuclear expression compared to control tumors [Fig. 2c] at day of sacrifice, while no significant differences were detected in the levels of apoptosis as indicated by cleaved caspase-3 immunostaining [Additional file 2: Figure S1C]. Together these data argue that, while a fraction of Bz-surviving cells may be sensitized to apoptosis even after drug-washout and this contributes to the time to recurrence, the long-term surviving fraction becomes deeply quiescent (or slow cycling) for variable periods of time after drug exposure. We further conclude that the PI-induced slow-cycling or quiescence is associated with a label-retaining CDK6^LOW^/p21^HIGH^ profile that was also previously reported by us (6).

### A Low-dose 5-aza-cytidine treatment potentiates Bz-induced cell death and deep quiescence

**Fig. 3:**
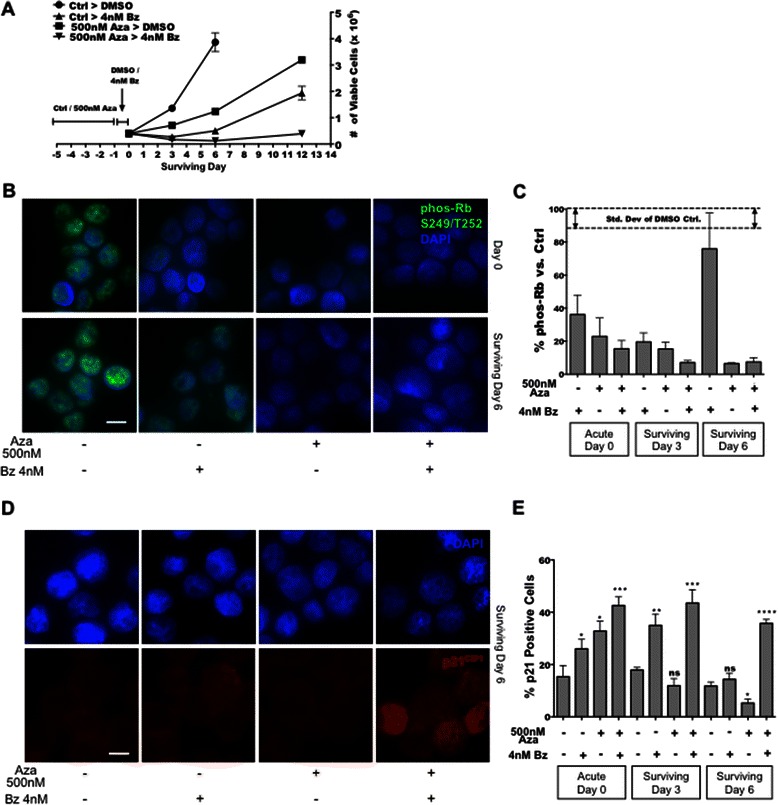
**a** Quantification of viable cells in Aza-reprogrammed (500 nM) Bz-surviving RPMI8226 cells compared to Bz only treatment, using Trypan blue exclusion assay. **b** & **c** IF detection and quantification of P-Rb (Ser249/Thr252) in RPMI8226 cells reprogrammed for 4 days ± 500 nM Aza, pulsed for 24 h with 4 nM Bz and stained 0, 3 (Additional file 3: Figure S2B) and 6 days after drug washout. **d** & **e** Detection and quantification of p21^CIP1^ in Aza-reprogrammed Bz-surviving RPMI8226 cells via IF. Scale bar =20 μm

In MM and other cancers the promoters of many tumor suppressors including p21^CIP1^, p15^INK4B^, p16^INK4A^ and apoptosis inducers are hypermethylated during transformation from monoclonal gammopathy of unknown significance (MGUS) to symptomatic MM [13]. Therefore, demethylating agents such as those used in the treatment of myelodysplastic syndromes (MDS) [14] may help restore the expression and response of these genes to stress signals. We next tested whether pre-treatment of MM cells with the DNA demethylating agent 5-aza-cytidine (Aza, 500 nM) might render MM cells more permissive for inducing a more long-lasting growth suppression after Bz treatment. MM cells were treated with Aza for 4 days followed by the 24 h Bz pulse and cell viability and phosphorylation of Rb (P-Rb) or expression of p21^CIP1^, Ki67, p15^INK4B^ and p16^INK4A^ were monitored. As reported, the Aza + Bz treatment enhanced the cell death induced by Bz [Additional file 3: Figure S2A] [15]. However, the viable surviving fraction after the Aza > Bz treatment displayed a significantly longer growth arrest (12 days), at least doubling the time observed with AZA alone or Bz alone treatments (4-6 days) [Fig. 3a]. We found that compared to DMSO controls, DMSO > Bz induced a 64 % decrease in P-Rb mean fluorescence intensity per cell by the end of the acute phase (Day 0) [Fig. 3b-c]. The Aza > DMSO and Aza > Bz-treated cells showed a 77 % and 85 % decrease in P-Rb levels, respectively. This reduction in P-Rb was more pronounced at 3 days for the Aza + Bz treatment [Additional file 3: Figure S2B]. Importantly, 6 days after the Aza > Bz treatment while Bz-only treated cells started to restore P-Rb levels, Aza > DMSO and Aza > Bz-treated cells continued to show 94 % and 93 % decrease, respectively [Fig. 3b-c]. This response was parelleled by sustained decrease in Ki67 levels [Additional file 3: Figure S2C-D] and suggested that Aza alone was sufficient to induce a growth arrest irrespectively of Bz treatment. However, at day 6, only the Aza > Bz treatment showed sustained high p21^CIP1^ [Fig. 3d-e] along with low levels of the proliferation markers (Ki67 and P-Rb) [Additional file 3: Figure S2C-D]. AZA > Bz showed slightly higher Ki67+ percent of cells than AZA > DMSO, which may indicate cells arrested in G1 (which still stains Ki67) while the AZA treatment might have the majority of cells in a G0. It is also possible that these differences might not be functionally relevant since p21^CIP1^ positive cells were more frequent and this may dominate the behavior of the population. Thus, the Aza > Bz treatment may be more effective in maintaining a strong p21-associated G0-G1 arrest. RPMI8226 and U266 cells showed a marginal benefit of Aza over the induction in p15^INK4B^, p16^INK4A^ and p21^cip1^mRNAs compared to Bz treatment alone [Additional file 3: Figure S2E]. This argues that the effects of Aza on p21 protein and cell cycle progression appear to be related to other changes in gene regulation and not simply CDKi mRNA upregulation.

### Upregulation of GRP78 in Bz-surviving MM cells is associated with disease progression in patients and therapy-mediated cell death

**Fig. 4:**
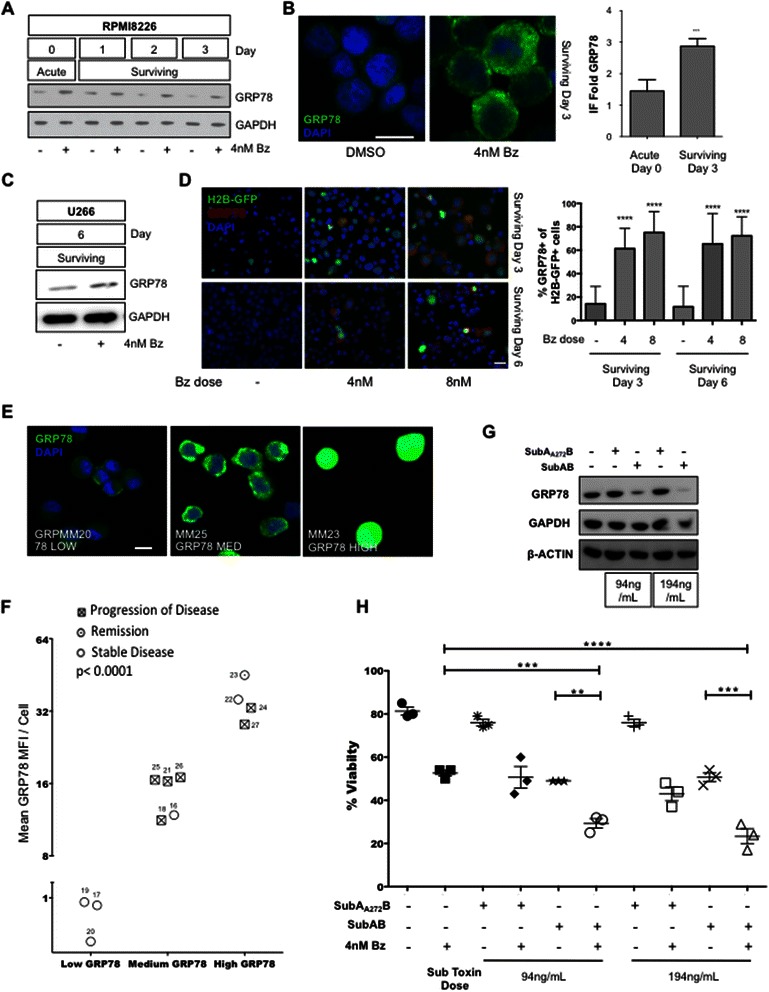
**a** Western blots for GRP78 protein in RPMI8226 cells. Due to abundance of GRP78 in RPMI8226 cells, 2 μg of protein per well (instead of 20 μg in other blots) was used in all blots. **b** IF Detection of GRP78 in Bz-surviving RPMI8226 cells. Fluorescence intensity quantification is shown as fold increase over the negative control. **c** Western blots for GRP78 protein in U266 cells 6 days after Bz washout. **d** Detection and quantification (right graph) of GRP78 in H2B-GFP^HIGH^ label-retaining cells at 3 and 6 days after drug washout by IF. **** p < 0.0001 (unpaired *t* test). Scale bar =25 μm. **e** Detection of GRP78 in cytospins from bone marrow aspirates of MM patients. Representatives of each group (low, medium, and high GRP78 levels) are shown here. Scale bar =20 μm. **f** Graphical representation of patient groups via GRP78 MFI per cell. Symbols represent stage of each patient. Patient MM# numbers are shown adjacent to each symbol. P < 0.0001 between groups (one-way ANOVA). **g** Western blots showing depletion of GRP78 protein in RPMI8226 cells after treatment with SubAB toxin. Non-functional mutant SubA_A272_B was used as a control. GAPDH and β-Actin were used as loading controls. **h** Cell viability plot of Bz-pulsed RPMI8226 cells, +/- GRP78 depletion via treatment (at two different concentrations) with SubAB toxin. Non-functional mutant SubA_A272_B was used as a control. Trypan blue exclusion was used as viability assay

We next explored whether GRP78, a UPR-induced gene upregulated in surviving residual MM cells during quiescence [6], was playing a role in their prolonged survival. We found that Bz-treated cells sustain the expression of GRP78 [Fig. 4a-b] and this induction was also confirmed in U266 cells even at 6 days post drug washout [Fig. 4c]. Importantly, the quiescent H2B-GFP label-retaining cells surviving 3 and 6 days after the Bz pulse showed a significant enrichment in GRP78 protein compared to control cells, as detected by IF [Fig. 4d]. This argues for a specific upregulation of GRP78 in quiescent cells upon proteasome inhibition. MG132-surviving RPMI 8226 cells also expressed more GRP78 suggesting that it is not a Bz-specific effect [Additional file 2: Figure S1B]. Q-PCR analysis of RPMI 8226 MM cells suggested that GRP78 mRNA was only induced in the acute phase (Day 0) and returned to basal levels in the Bz-surviving cells [Additional file 4: Figure S3A] arguing for a post-transcriptional regulation of the protein in Bz-surviving cells, possibly through the 5′ internal ribosome entry site on GRP78 mRNA [16].

GRP78 is a well-characterized survival factor across different cancers and in both proliferative and quiescent states [6, 17, 18]. To provide a preliminary assessment of the possibility that GRP78 may be used as a survival marker in MM cells from patients, we measured the levels of GRP78 in CD138+ sorted cells isolated from BM aspirates from 12 patients. Control experiments confirmed an almost complete enrichment of CD138^HIGH^ cells after magnetic bead separation [Additional file 4: Figure S3B-D]. These isolated cells were prepared in cytospins, stained for GRP78 [Fig. 4e] and the GRP78 MFI was quantified. Intensities were normalized to the fluorescence intensities of controls. Bearing in mind that our study is a small sample size, we found that using the MFI data values, patient samples separated into 3 groups, low (<1 GRP78 MFI arbitrary units), medium (1-24 GRP78 MFI) and high (>24 GRP78 MFI) based on calculated median levels of GRP78 MFI levels/cell. All three GRP78^LOW^ patients (25 %) were characterized as stable disease at time of sample collection. The nine patients with medium and high GRP78 (75 %) had progressive disease, while within these two subcategories 2 patients had stable disease (22 %) and one (11 %) was in remission [Table 1]. Grouping by response criteria showed that 60 % of patients with stable disease had GRP78^LOW^ MM cells and 100 % with progressive disease had CD138+ cells with medium or high GRP78 levels [Fig. 4f]. Taking into account the limitations of sample size and heterogeneity in treatment, these data could be interpreted as GRP78^HIGH^ levels contributing to a MM survival advantage. GRP78^MEDIUM^ and GRP78^HIGH^ levels in CD138+ cells in two patients with stable disease or in remission might represent MM cells that are slow or non-proliferative but with a high survival advantage.

**Table 1:** Characteristics for the patients whose BM samples were tested for BiP levels in CD138+ cells

MM code	Age at diagnosis	Ouant Immunoglobulins and Serum immunofixation at collection	Albumin	LDH	Prior Bortezomib?	Myeloma status at time of Collection
MM16	64	lgG 2371, monoclonal protein in gamma region	4.3	201	no	Stable disease
MM17	59	IgG 282, IgA 5083, IgM 16, Two IgA lambda monoclonal band seen, representing 85 %	3.7	106	yes	Stable disease
MM18	68	IgG 2333, IgA 12, 1gM 20, monoclonal protein seen in gamma region	3.3	175	yes	Progression of Disease (increase in M-spike)
MM19	46	IgG 4656, IgA 27, 1 gM 53, monoclonal protein in gamma region	3.8	142	no	Minimal response/stable disease
MM20	unav	IgG 4099, monoclonal band in gamma region	3.7	121	unav	Stable Disease
MM21	56	IgG 314, 1 gM <5, IgA <5, faint free kappa band	4.7	143	yes	Progression of Disease
MM22	52	IgG 453, 1 gM 7, IgA 132, faint IgG lambda band	2.7	170	yes	partial response/stable disease
MM23	70	lgG 840, IgA 9, 1 gM 9, lgG kappa monoclonal spike seen, representing all of monoclonal protein	4.5	564	yes	Remission (very good partial response)
MM24	57	lgG 255, 1 gM 12, IgA 19, faint free Lambda band seen	3.1	154	yes	Progression of Disease
MM25	39	lgG 188, IgA 10, 1gM 12, Free monoclonal lambda light chain, normal Igs greatly diminished	4.7	601	yes	Progression of Disease
MM26	66	lgG 6589, Iga 8, 1 gM <5, lgG kappa monoclonal representing all of total	3.9	166	yes	Progression of Disease
MM27	54	lgG 5543, IgA 11, 1 gM 19, lgG kappa monoclonal protein	4.1	210	yes	Progression of Disease

We next depleted the GRP78 protein to assess whether cells hypomorphic for this chaperone were unable to survive Bz-induced cell death. To this end, we used subtilase cytotoxin (SubAB), a bacterial AB_5_ toxin that has been shown to specifically cleave GRP78 (18, 19) [Fig. 4g]. As a control, we used a non-functional mutant toxin termed SubA_A272_B. Inhibition of GRP78 using the IC_50_ for SubAB significantly decreased the viability of the MM cells after Bz treatment compared to the non-functional mutant SubA_272_B [Fig. 4h]. This suggests that GRP78 is a major survival factor in residual Bz-surviving cells and a potential target to eradicate these residual cells.

## Discussion

Multiple myeloma cells synthesize and secrete large amounts of immunoglobulins [19] and thus possess a very tightly regulated ER quality control system. The proteasome inhibitor bortezomib was the first in its class to be FDA-approved for treatment of MM patients [20, 21] and second generation agents are now also available [22]. While proteasome inhibition is a standard of care for MM, patients invariably relapse. This suggests that a small fraction of neoplastic cells can escape this treatment through poorly understood mechanisms. We hypothesized that by exploring the biology of the residual surviving MM cells we may identify markers for residual cells and survival mechanisms to target and prevent MM relapse.

We had previously found that Bz-surviving MM cells entered quiescence and silenced specific components of the UPR signaling that commonly induce cell death [6, 17, 23, 24]. However, what genes may mark quiescent cells with enhanced survival properties and what components of the UPR might promote survival was unknown. Here we show that after a Bz pulse, the residual cells are for the most part slow-cycling as expected by the growth arrest propelled by high eIF2α phosphorylation [25]. However, we also found a deeply quiescent and viable fraction of cells that were marked by p21^HIGH^ levels and prolonged H2B-GFP label retention. In addition to p21 upregulation, which appeared to be transcriptional, Bz-surviving MM cells showed loss of CDK6 and consequently loss of P-Rb protein [6], which could explain the G0-G1 cell cycle arrest in MM cells. These data argue that while slow-cycling is a main response to Bz, a small fraction is capable of entering a deeper quiescence. Importantly, these cells were preferentially enriched for GRP78 arguing they may be prone to enhanced survival. It is possible that with repeated cycles of PI treatments used in the clinic more of the deep quiescent MM cells that survive the treatment accumulate creating a population that escapes Bz treatment and anti-proliferative drugs, eventually fueling relapses. That these cells may become “professional” ER stress tolerant is suggested by the upregulation of GRP78 that was also found in MM cells from patients with progressive disease. Our in vivo data using U266 MM cells argues that p21^HIGH^ MM cells can be found and may persist without expanding for ~90 days (~1 year in humans) after a 24 h pulse with Bz. The lack of apoptosis in these dormant lesions and the upregulation of p21 coupled to no net increase in tumor mass argues against continuous apoptosis and in favor of long-term quiescence as a mechanism to explain de prolonged time to take of these MM cells in vivo. We propose that in the bone marrow of patients a specific MM cell subpopulation (CDK6^LOW^/P-Rb^LOW^/p21^HIGH)^ may be found dormant after Bz treatment.

Many common quiescence regulators such as the tumor suppressors p15^INK4B^ [26] and p16^INK4A^ are epigenetically silenced in cancer [27]. Our data shows that mRNA induction of p15^INK4B^, p16^INK4A^ and p21^CIP1^ (and protein) in surviving MM cells is not greatly increased by an Aza pre-treatment and Bz pulse. However, the initial apoptosis and later prolonged growth arrest phase in vitro is more than doubled in cells treated with Aza and Bz and this correlated with p21^CIP1^, Ki67 and P-Rb levels in viable growth-arrested cells. While we have not performed detailed gene promoter methylation analysis to determine the targets influenced by the Aza treatment, our data suggested that “reprogramming” with Aza might be amenable to be used as a way to maximizing the apoptosis but also quiescence induction effects of Bz.

Our work also tested the role of GRP78, a well-characterized survival component of the UPR [28] that is upregulated and promotes drug resistance of quiescent squamous cell carcinoma (HNSCC) cells [17]. Here we found that Bz-surviving and quiescent (viable H2B-GFP label- retaining) MM cells maintained high levels of GRP78 for many days after drug washout, arguing these quiescent cells may selectively upregulate this ER chaperone. This suggests that GRP78 is important for cell survival during PI-mediated UPR activation in the quiescent MM cell population. Importantly, targeted depletion of GRP78 enhanced Bz-mediated cell death, justifying further studies to test if this chaperone might be an amenable therapeutic target in the resistant residual disease. Overexpression of GRP78 was correlated with clinical progression in other cancer models [29–31]. We found GRP78 upregulation might be associated to disease progression in MM patient samples. Because our patient sample size is small, we cautiously propose that either in residual MM or recurrent MM cells, GRP78 is likely to mark a subpopulation with enhanced survival. Our analysis of patient samples was a pilot study and larger cohorts of patients tested for GRP78 expression in their MM samples may prove useful to determine whether this chaperone of the ER is indeed a marker to distinguish persistent Bz-refractory and/or recurrent disease.

## Conclusions

We conclude that Bz-surviving MM cells display a GRP78^HIGH^/p21^HIGH^/CDK6^LOW^/P-Rb^LOW^ profile. These markers may pinpoint quiescent MM cells capable of fueling recurrences. We further conclude that upregulation of GRP78 allows specifically quiescent tumor cells to survive for prolonged periods and this may be an amenable target to kill residual MM cells. Although the mechanisms are incompletely understood, we also conclude that the combination of Aza and Bz treatments may represent a novel strategy to delay MM recurrences by enhancing Bz-induced apoptosis and the stability of the quiescence program.

## Additional files

Additional file 1: Table S1. List of primer sequences used in the study.
Additional file 2: Figure S1. [**A**] Quantification of viable U266 cells PI-pulsed (MG132 400nM) using trypan blue exclusion test. [**B**] Western blots for CDK4, CDK6, Cyclin D1, Cyclin D3, and GRP78 protein in MG132-surviving RPMI8226 cells. GAPDH was used as a loading control. [**C**] IHC detection of cleaved caspase 3 levels in tumors derived from RPMI8226 and U266 cells pulsed for 24h with DMSO or 8nM Bz (n = 3). Quantification of percentage of cleaved caspase 3 positive cells per tumor sections. * p = n.s comparing DMSO vs. 8nM Bz (unpaired *t* test). Scale bar =25 μm.
Additional file 3: Figure S2. [**A**] Viability of RPMI8226 cells at Day 0 and Day 3 after Bz pulse, with or without Aza pre-treatment (500 nM) as determined by Trypan blue exclusion test. [**B**] IF detection of P-Rb (Ser249/Thr252) in RPMI8226 cells reprogrammed for 4 days ± 500 nM Aza, pulsed for 24 h with 4 nM Bz and stained 3 days after drug washout. [**C**] IF detection and [**D**] quantification of Ki67 in RPMI8226 cells reprogrammed for 4 days ± 500 nM Aza, pulsed for 24 h with 4 nM Bz and stained 6 days after drug washout. [**E**] qRT-PCR for p15, p16 and p21 mRNA expression in RPMI8226 and U266 cells after Bz pulse, with or without Aza pre-treatment. The mRNA levels were normalized with tubulin expression.
Additional file 4: Figure S3. [**A**] qRT-PCR showing fold increase in GRP78 mRNA expression in Bz-surviving RPMI8226 cells 0, 3 and 6 days after drug washout. The mRNA levels were normalized to GAPDH and then compared to DMSO controls. [**B**] Detection and [**C**] quantification plot of CD138 in MM patient bone marrow aspirates before and after magnetic beads-based purification of CD138-positive cells. Staining for CD138 in RPMI8226 cells (right panel) was used as a positive control. [**D**] Representative images of CD138 purification in MM patient samples. Scale bar =20 μm.

## References

[1] Chauhan D, Singh A, Brahmandam M, Podar K, Hideshima T, Richardson P (2008). Combination of proteasome inhibitors bortezomib and NPI-0052 trigger in vivo synergistic cytotoxicity in multiple myeloma. Blood.

[2] Laubach JP, Mahindra A, Mitsiades CS, Schlossman RL, Munshi NC, Ghobrial IM (2009). The use of novel agents in the treatment of relapsed and refractory multiple myeloma. Leukemia.

[3] Lee HC, Shah JJ, Orlowski RZ (2013). Novel approaches to treatment of double-refractory multiple myeloma. Am Soc Clin Oncol Educ Book.

[4] McMillin DW, Jacobs HM, Delmore JE, Buon L, Hunter ZR, Monrose V (2012). Molecular and cellular effects of NEDD8-activating enzyme inhibition in myeloma. Mol Cancer Ther.

[5] Badros AZ (2010). The role of maintenance therapy in the treatment of multiple myeloma. J Natl Compr Canc Netw.

[6] Schewe DM, Aguirre-Ghiso JA (2009). Inhibition of eIF2alpha dephosphorylation maximizes bortezomib efficiency and eliminates quiescent multiple myeloma cells surviving proteasome inhibitor therapy. Cancer Res.

[7] Flanders A, Stetler-Stevenson M, Landgren O (2013). Minimal residual disease testing in multiple myeloma by flow cytometry: major heterogeneity. Blood.

[8] Rajkumar SV, Richardson PG, Hideshima T, Anderson KC (2005). Proteasome inhibition as a novel therapeutic target in human cancer. J Clin Oncol.

[9] Massey AJ, Williamson DS, Browne H, Murray JB, Dokurno P, Shaw T (2010). A novel, small molecule inhibitor of Hsc70/Hsp70 potentiates Hsp90 inhibitor induced apoptosis in HCT116 colon carcinoma cells. Cancer Chemother Pharmacol.

[10] Aguirre Ghiso JA, Kovalski K, Ossowski L (1999). Tumor dormancy induced by downregulation of urokinase receptor in human carcinoma involves integrin and MAPK signaling. J Cell Biol.

[11] Schaniel C, Moore KA (2009). Genetic models to study quiescent stem cells and their niches. Ann N Y Acad Sci.

[12] Wilson A, Laurenti E, Oser G, van der Wath RC, Blanco-Bose W, Jaworski M (2008). Hematopoietic stem cells reversibly switch from dormancy to self-renewal during homeostasis and repair. Cell.

[13] Sharma A, Heuck CJ, Fazzari MJ, Mehta J, Singhal S, Greally JM (2010). DNA methylation alterations in multiple myeloma as a model for epigenetic changes in cancer. Wiley Interdiscip Rev Syst Biol Med.

[14] Silverman LR, Fenaux P, Mufti GJ, Santini V, Hellstrom-Lindberg E, Gattermann N (2011). Continued azacitidine therapy beyond time of first response improves quality of response in patients with higher-risk myelodysplastic syndromes. Cancer.

[15] Kiziltepe T, Hideshima T, Catley L, Raje N, Yasui H, Shiraishi N (2007). 5-Azacytidine, a DNA methyltransferase inhibitor, induces ATR-mediated DNA double-strand break responses, apoptosis, and synergistic cytotoxicity with doxorubicin and bortezomib against multiple myeloma cells. Mol Cancer Ther.

[16] Yang Q, Sarnow P (1997). Location of the internal ribosome entry site in the 5′ non-coding region of the immunoglobulin heavy-chain binding protein (BiP) mRNA: evidence for specific RNA-protein interactions. Nucleic Acids Res.

[17] Ranganathan AC, Zhang L, Adam AP, Aguirre-Ghiso JA (2006). Functional coupling of p38-induced up-regulation of BiP and activation of RNA-dependent protein kinase-like endoplasmic reticulum kinase to drug resistance of dormant carcinoma cells. Cancer Res.

[18] Dong D, Dubeau L, Bading J, Nguyen K, Luna M, Yu H (2004). Spontaneous and controllable activation of suicide gene expression driven by the stress-inducible grp78 promoter resulting in eradication of sizable human tumors. Hum Gene Ther.

[19] Eslick R, Talaulikar D (2013). Multiple myeloma: from diagnosis to treatment. Aust Fam Physician.

[20] Richardson PG, Barlogie B, Berenson J, Singhal S, Jagannath S, Irwin D (2003). A phase 2 study of bortezomib in relapsed, refractory myeloma. N Engl J Med.

[21] Bross PF, Kane R, Farrell AT, Abraham S, Benson K, Brower ME (2004). Approval summary for bortezomib for injection in the treatment of multiple myeloma. Clin Canc Res.

[22] Herndon TM, Deisseroth A, Kaminskas E, Kane RC, Koti KM, Rothmann MD (2013). U.s. Food and Drug Administration approval: carfilzomib for the treatment of multiple myeloma. Clin Canc Res.

[23] Aguirre-Ghiso JA, Estrada Y, Liu D, Ossowski L (2003). ERK(MAPK) activity as a determinant of tumor growth and dormancy; regulation by p38(SAPK). Cancer Res.

[24] Schewe DM, Aguirre-Ghiso JA (2008). ATF6alpha-Rheb-mTOR signaling promotes survival of dormant tumor cells in vivo. Proc Natl Acad Sci U S A.

[25] Brewer JW, Diehl JA (2000). PERK mediates cell-cycle exit during the mammalian unfolded protein response. Proc Natl Acad Sci U S A.

[26] Seidl S, Ackermann J, Kaufmann H, Keck A, Nosslinger T, Zielinski CC (2004). DNA-methylation analysis identifies the E-cadherin gene as a potential marker of disease progression in patients with monoclonal gammopathies. Cancer.

[27] Chim CS, Fung TK, Liang R (2003). Disruption of INK4/CDK/Rb cell cycle pathway by gene hypermethylation in multiple myeloma and MGUS. Leukemia.

[28] Hendershot LM (2004). The ER function BiP is a master regulator of ER function. Mount Sinai J Med New York.

[29] Zhuang L, Scolyer RA, Lee CS, McCarthy SW, Cooper WA, Zhang XD (2009). Expression of glucose-regulated stress protein GRP78 is related to progression of melanoma. Histopathology.

[30] Kuroda K, Horiguchi A, Asano T, Ito K, Asakuma J, Sato A (2011). Glucose-regulated protein 78 positivity as a predictor of poor survival in patients with renal cell carcinoma. Urol Int.

[31] Thornton M, Aslam MA, Tweedle EM, Ang C, Campbell F, Jackson R (2013). The unfolded protein response regulator GRP78 is a novel predictive biomarker in colorectal cancer. Int J Canc.

