# Supplementary figures and images for: Identification of markers that functionally define a quiescent multiple myeloma cell sub-population surviving bortezomib treatment
Source: BMC Cancer. 2015 May 30;15:444. doi: 10.1186/s12885-015-1460-1 (PMC4448210; doi:10.1186/s12885-015-1460-1)

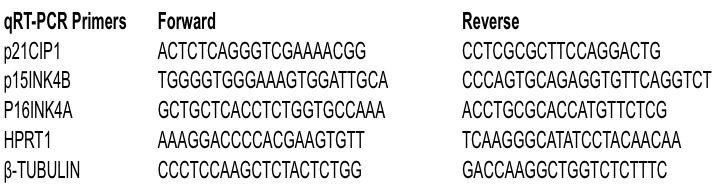

Supplement: Additional file 1: Table S1. — List of primer sequences used in the study. [file 12885_2015_1460_MOESM1_ESM.tiff]

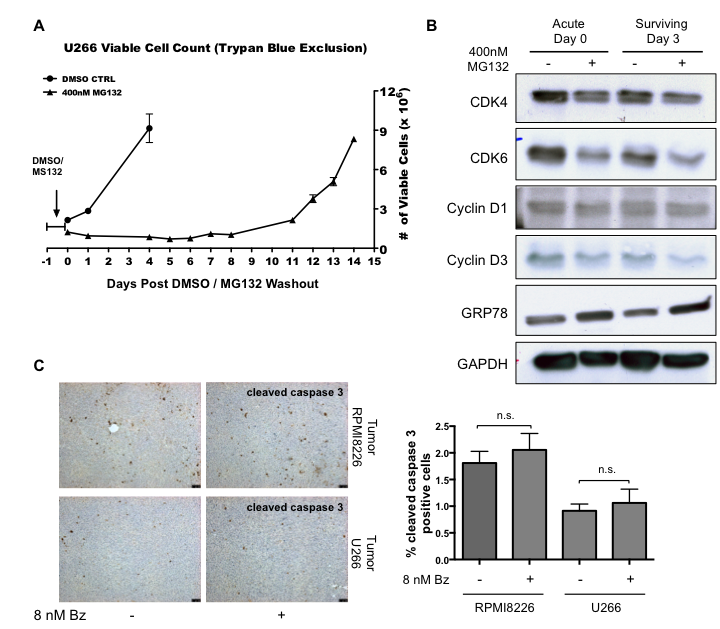

Supplement: Additional file 2: Figure S1. — [A] Quantification of viable U266 cells PI-pulsed (MG132 400nM) using trypan blue exclusion test. [B] Western blots for CDK4, CDK6, Cyclin D1, Cyclin D3, and GRP78 protein in MG132-surviving RPMI8226 cells. GAPDH was used as a loading control. [C] IHC detection of cleaved caspase 3 levels in tumors derived from RPMI8226 and U266 cells pulsed for 24h with DMSO or 8nM Bz (n = 3). Quantification of percentage of cleaved caspase 3 positive cells per tumor sections. * p = n.s comparing DMSO vs. 8nM Bz (unpaired t test). Scale bar =25 μm. [file 12885_2015_1460_MOESM2_ESM.png]

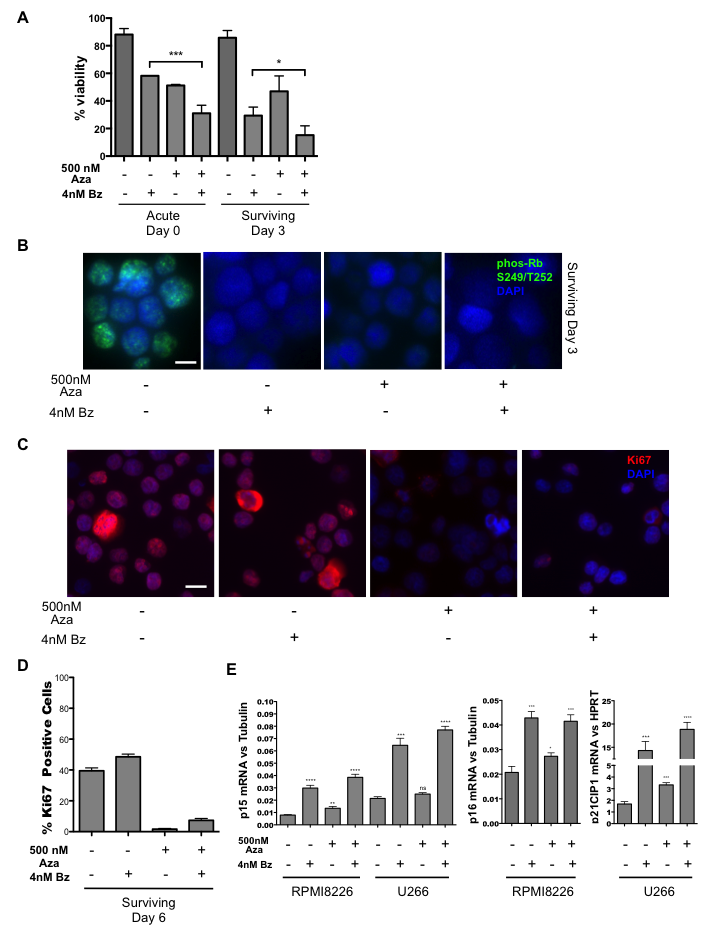

Supplement: Additional file 3: Figure S2. — [A] Viability of RPMI8226 cells at Day 0 and Day 3 after Bz pulse, with or without Aza pre-treatment (500 nM) as determined by Trypan blue exclusion test. [B] IF detection of P-Rb (Ser249/Thr252) in RPMI8226 cells reprogrammed for 4 days ± 500 nM Aza, pulsed for 24 h with 4 nM Bz and stained 3 days after drug washout. [C] IF detection and [D] quantification of Ki67 in RPMI8226 cells reprogrammed for 4 days ± 500 nM Aza, pulsed for 24 h with 4 nM Bz and stained 6 days after drug washout. [E] qRT-PCR for p15, p16 and p21 mRNA expression in RPMI8226 and U266 cells after Bz pulse, with or without Aza pre-treatment. The mRNA levels were normalized with tubulin expression. [file 12885_2015_1460_MOESM3_ESM.tiff]

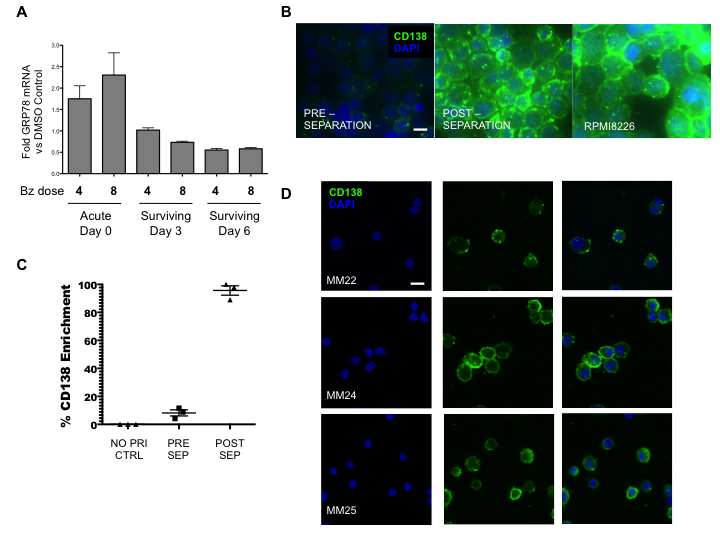

Supplement: Additional file 4: Figure S3. — [A] qRT-PCR showing fold increase in GRP78 mRNA expression in Bz-surviving RPMI8226 cells 0, 3 and 6 days after drug washout. The mRNA levels were normalized to GAPDH and then compared to DMSO controls. [B] Detection and [C] quantification plot of CD138 in MM patient bone marrow aspirates before and after magnetic beads-based purification of CD138-positive cells. Staining for CD138 in RPMI8226 cells (right panel) was used as a positive control. [D] Representative images of CD138 purification in MM patient samples. Scale bar =20 μm. [file 12885_2015_1460_MOESM4_ESM.tiff]
